# HLA-Cw*0602 associates with a twofold higher prevalence of positive streptococcal throat swab at the onset of psoriasis: a case control study

**DOI:** 10.1186/1471-5945-9-5

**Published:** 2009-05-29

**Authors:** Lotus Mallbris, Katarina Wolk, Fabio Sánchez, Mona Ståhle

**Affiliations:** 1 Dermatology Unit, Department of Medicine, Karolinska Institutet, Stockholm, Sweden

## Abstract

**Background:**

The influence of streptococcal infections in the pathogenesis of psoriasis is not yet understood. *In vitro *data suggest that streptococcal factors influence T-cell function in psoriasis in a HLA-dependent manner, but studies designed to measure the HLA-C/Streptococci interaction are lacking. In the present study, we hypothesized that there is a statistical interaction between the result of streptococcal throat cultures and the presence of the HLA-Cw*0602 allele in psoriasis patients.

**Methods:**

We performed a case control study using the "Stockholm Psoriasis Cohort" consisting of patients consecutively recruited within 12 months of disease onset (Plaque psoriasis = 439, Guttate psoriasis = 143), matched to healthy controls (n = 454) randomly chosen from the Swedish Population Registry. All individuals underwent physical examination including throat swabs and DNA isolation for HLA-Cw*0602 genotyping.

The prevalence of positive streptococcal throat swabs and HLA-Cw*0602 was compared between patients and controls and expressed as odds ratios with 95% confidence intervals. Associations were evaluated separately for guttate and plaque psoriasis by Fisher's exact test.

**Results:**

Regardless of disease phenotype, the prevalence of positive streptococcal throat swabs in HLA-Cw*0602 positive patients was twice the prevalence among HLA-Cw*0602 negative patients (OR = 5.8 C.I. = 3.57–9.67, p < 0.001), while no difference was observed among Cw*0602 positive versus negative controls.

The corresponding odds ratios for the guttate and plaque psoriasis phenotypes were 3.5 (CI = 1.5–8.7, p = 0.01) and 2.3 (CI = 1.0–5.1, p = 0.02) respectively.

**Conclusion:**

These findings suggest that among HLA-Cw*0602 positive psoriasis patients, streptococci may contribute to the onset or exacerbation of the inflammatory process independent of the disease phenotype. However, studies on the functional interaction between HLA-C and streptococcal factors are needed.

## Background

Psoriasis is phenotypically and genetically heterogeneous and associates with various environmental triggers. Gene-environment interactions are relevant for complex diseases [1,2]; for example, smoking influences seropositive rheumatoid arthritis in an HLA-DR-dependent manner [3]. In psoriasis, so far, only circumstantial evidence links streptococcal infections to HLA-Cw*0602 status, mainly in the guttate phenotype [4-8]. HLA genotypes are reported to influence T-cell proliferation in response to streptococcal antigens *in vitro *[9,10] and streptococci are known to modulate immune responses through molecular mimicry [11]. *In vitro *studies show homology between streptococcal M-proteins and human keratins, which may influence T-cell responses [10]. However, assessment of associations between streptococcal infection and HLA-Cw6 at the population level is needed [12-18]. The present work addresses this by investigating the prevalence of positive streptococcal throat swabs in guttate (GP) and plaque psoriasis (PP) in relation to HLA-Cw*0602 status.

## Methods

### Study subjects

Patients from the "Stockholm Psoriasis Cohort" (PP = 439, GP = 143) were consecutively recruited within 12 months of disease onset [15]. Psoriasis was diagnosed according to established criteria [19]. Matched healthy controls (n = 454) were randomly chosen from the Swedish Population Registry. All individuals underwent physical examination including throat swab. To account for seasonal variations, throat swabs from matched cases and controls were obtained at the same time of the year. Blood samples were obtained for DNA extraction. Written informed consent was given by all individuals. The study was approved by the Regional Committee of Ethics and performed according to the Declaration of Helsinki Principles.

### HLA-Cw*0602 genotyping

Genotyping was performed by allele specific PCR amplification of HLA-Cw*0602 as described [20].

### Statistical Analyses

The prevalence of positive streptococcal throat swabs and HLA-Cw*0602 was compared between patients and controls and expressed as odds ratios with 95% confidence intervals. Associations were evaluated separately for GP and PP by Fisher's exact test. Analyses were performed using the Statistical Analysis System package [21].

## Results

### Demographic and clinical characteristics

Age at onset ranged from 14–84 years, with a mean age of 32 for GP, 44 for PP and 40 for controls. The sex distribution was slightly skewed with a female predominance (controls: 60%, GP: 58% and PP: 54%). Overall, 2/3 of patients had mild psoriasis. The mean PASI-score was 5.6 for GP and 3.7 for PP. The delay time between the first psoriasis lesion and clinical examination was 4.0 months for GP and 7.5 months for PP.

### Streptococcal throat status

All bacterial strains isolated from patients were β-hemolytic *Streptococcus pyogenes*, group A (76%), C (8%) and G (16%). In controls, no group A streptococci were isolated, 7 individuals had group G and one had group C. Positive streptococcus isolation was 7.9 and 27.5 times more prevalent in GP patients compared to PP and controls respectively (Table 1).

**Table 1:** HLA-Cw*0602 and streptococcal status

		Positive		Negative		OR^a^		
					
		N	%	N	%	OR	95% CI^b^	*p*
					
HLA-Cw*0602								
Guttate	(n = 143)	106	74	37	26	23.7	14.3–39.3	<0.001
Plaque	(n = 439)	134	31	305	69	3.6	2.5–5.3	<0.001
Control	(n = 454)	49	11	405	89			
								
**Streptococcus throat swab**								
Guttate	(n = 143)	79	55	64	45	68.8	31–170	<0.001
Plaque	(n = 439)	31	7	408	93	4.2	1.9–10.8	<0.001
Control	(n = 454)	8	2	446	98			

^a^OR = Odds Ratio (Patients compared to the healthy control)

^b^CI = confidence interval

### HLA-Cw*0602 associates strongly with GP

Regardless of disease phenotype, there were 240 HLA-Cw*0602 positive and 342 negative patients and 49 HLA-Cw*0602 positive and 405 negative controls (Table 1).

HLA-Cw*0602 was more frequent in GP (74%) compared with PP (31%) and controls (11%), (Table 1).

### Twofold higher prevalence of positive streptococcal throat swabs in HLA-Cw*0602 positive psoriasis patients

In absolute numbers, there were significantly more GP patients with a positive streptococcal culture who carried HLA-Cw*0602 (67/143), compared to PP (15/439). However, in both groups, the prevalence of positive streptococcal cultures increased approximately twofold among Cw*0602 positive patients (GP: ×1.97; PP: ×2.2), while no difference was observed among controls (Table 2).

**Table 2:** Interaction between HLA-Cw*0602 and streptococcal status

		-/+		+/+		OR^a^		
					
		N	%	N	%	OR	95% CI	*p*
					
HLA-Cw*0602/Streptococci								
Guttate	(n = 143)	12	32	67	63	3.5	1.5–8.7	<0.01
Plaque	(n = 439)	16	5	15	11	2.3	1.0–5.1	<0.02
Control	(n = 454)	7	2	1	2	1.2	0.02–9.7	0.6

^a^OR = Odds Ratio (HLA-Cw*0602 positive individuals compared to HLA-Cw*0602 negative individuals)

^b^CI = confidence interval

## Discussion

A link between streptococcal infections and several autoimmune disorders has been observed, the clearest being cardiac and renal damage due to cross-reactivity with streptococcal antigens [22,23]. Interestingly, a recent study showed that the global epidemiological variation in psoriasis prevalence correlates closely with that of historical mortality from epidemics of invasive streptococcal infections. The study suggested that the psoriasis genotype could confer a degree of protection from mortality in such epidemics [24]. In psoriasis, numerous studies suggest a role for streptococcal factors in triggering disease, particularly for GP [13,15,25,26]. It has been proposed that psoriasis may be due to autoimmunity resulting from cross-reaction between streptococcal and skin epitopes, such as streptococcal M protein and keratin [27], or cytokines released by the superantigen stimulated T cells [28]. Also, peptidoglycan (PG), the major constituent of the streptococcal cell wall, has been suggested to acts as a T cell activator in psoriasis [29].

Since psoriasis has a strong genetic basis, associating to HLA-Cw*0602 [30], and since this molecule has an important immunological role an interaction between HLA-Cw*0602 and streptococci has been suggested [31]. The present study hypothesized that in the initial stages of disease, the prevalence of streptococcus positive cultures is higher among HLA-Cw*0602 positive psoriasis patients.

Consistent with previous reports, our results demonstrate that HLA-Cw*0602 is more prevalent among young patients and in GP [32,33]. Also, in accordance with other reports, we observed that streptococcal throat infection was more common in GP at disease onset [13,17]. This could be partly explained by earlier medical attention for GP, due to the acuteness of the disease, compared to PP. In this study, the time between disease onset and streptococcus swabs was variable. In most cases culture for streptococcus was performed a few weeks after the onset of psoriasis. Thus, the presence of streptococcus may in some cases be unrelated to psoriasis. However, the fact that after accounting for seasonal variations, positive cultures were significantly more prevalent among patients than controls indicates that these factors are likely related.

The main finding of the study is the twofold increased prevalence of positive streptococcal throat swabs among HLA-Cw*0602 positive patients compared to negative patients, regardless of disease phenotype, suggesting that the interaction between HLA-Cw*0602 and streptococci modulate disease independent of clinical subtype. As expected, the frequency of positive streptococcal cultures among healthy controls was low (2%), indicating that carrying HLA-Cw*0602 does not increase the risk of streptococcal throat infection.

Even though several groups studied HLA-Cw*0602 and streptococcal infection independently [12-16], only one Irish study investigated their interaction in 64 patients with GP, 41 patients with PP and 20 controls [17]. They concluded that HLA-Cw*0602 is not essential for streptococcal-induced psoriasis. However, it is feasible that different genetic factors influence the expression of HLA-Cw*0602 and the prevalence of streptococci throat infection among different population. Still, these factors may contribute to onset of psoriasis.

Compared with other studies in Caucasians, the prevalence of HLA-Cw*0602 positive individuals in the Irish study was surprisingly high among PP patients (73%) and among controls (18%) [17]. However, it is conceivable that the higher prevalence of HLA-Cw6 in the general population reflects a higher prevalence of psoriasis in that population.

Neither HLA-Cw*0602, nor streptococcal infections *per se *seem necessary for disease development, because there are psoriasis patients that do not carry HLA-Cw*0602 and develop disease without signs of streptococcal infection. It is possible that other HLA-C alleles may substitute for Cw*0602 when interacting with streptococci, and also, other triggering factors besides streptococci may contribute to disease through HLA-Cw*0602.

## Conclusion

There is growing awareness that the clinical subtypes of psoriasis may have different genetic backgrounds, which has implications for study design when trying to measure gene/environment associations [34]. Yet, we observed that the HLA-Cw*0602/streptococci interaction was independent of disease phenotype, even though the absolute numbers were small among plaque patients. It is still unclear how this combination of factors influences the expression of clinical manifestations of psoriasis, therefore functional assessment of HLA-Cw*0602/streptococci interaction is required.

## List of abbreviations

CI: Confidence intervals; GP: Guttate psoriasis; HLA-C: Human Leukocyte Antigen C; OR: Odds Ratio; PASI: Psoriasis Area and Severity Index; PP: Plaque psoriasis; PSORS: Psoriasis Susceptibility Locus.

## Competing interests

The authors declare that they have no competing interests.

## Authors' contributions

LM participated in the design of the study, collected most of the data, performed all statistical analysis, interpreted the data, as well as drafted the manuscript. KW participated in the data collection. FS participated in the design of the study, carried out the genotyping, interpreted the data and contributed to the manuscript writing. MS participated in the design of the study, interpreted the data, contributed to the manuscript writing and provided funding. All authors read and approved the final manuscript.

## Pre-publication history

The pre-publication history for this paper can be accessed here:


